# The janus-kinase inhibitor ruxolitinib in SARS-CoV-2 induced acute respiratory distress syndrome (ARDS)

**DOI:** 10.1038/s41375-021-01374-3

**Published:** 2021-08-12

**Authors:** Andreas Neubauer, Johannes Johow, Elisabeth Mack, Andreas Burchert, Damaris Meyn, Andrea Kadlubiec, Iuliu Torje, Hinnerk Wulf, Claus F. Vogelmeier, Joachim Hoyer, Chrysanthi Skevaki, Ralf Michael Muellenbach, Christian Keller, Carmen Schade-Brittinger, Caroline Rolfes, Thomas Wiesmann

**Affiliations:** 1 Klinik für Innere Medizin, Hämatologie, Onkologie, Immunologie, Philipps Universität and UKGM, Marburg, Germany; 2 Coordinating Center for Clinical Trials, Philipps Universität, Marburg, Germany; 3 Apotheke, Klinikum Kassel, Kassel, Germany; 4 Klinik für Anästhesiologie und Intensivmedizin, Klinikum Kassel, Kassel, Germany; 5 Klinik für Anästhesiologie und Intensivmedizin, Philipps Universität and UKGM, Marburg, Germany; 6 Klinik für Innere Medizin, Schwerpunkt Pneumologie, Intensiv- und Schlafmedizin, Philipps Universität and UKGM, Member of the German Center for Lung Research (DZL), Marburg, Germany; 7 Klinik für Innere Medizin, Nephrologie, Philipps Universität and UKGM, Marburg, Germany; 8 Institut für Labormedizin, Universities of Giessen and Marburg Lung Center (UGMLC), Philipps Universität Marburg, German Center for Lung Research (DZL), Marburg, Germany; 9 Institut für Virologie, Philipps Universität and UKGM, Marburg, Germany

**Keywords:** Infectious diseases, Phase II trials

## Abstract

Severe acute respiratory syndrome coronavirus 2 (SARS-CoV-2) causes COVID-19 (coronavirus disease 2019), which is associated with high morbidity and mortality, especially in elder patients. Acute respiratory distress syndrome (ARDS) is a life-threatening complication of COVID-19 and has been linked with severe hyperinflammation. Dexamethasone has emerged as standard of care for COVID-19 associated respiratory failure. In a non-randomized prospective phase II multi-center study, we asked whether targeted inhibition of Janus kinase-mediated cytokine signaling using ruxolitinib is feasible and efficacious in SARS-CoV-2- induced ARDS with hyperinflammation. Sixteen SARS-CoV-2 infected patients requiring invasive mechanical ventilation for ARDS were treated with ruxolitinib in addition to standard treatment. Ruxolitinib treatment was well tolerated and 13 patients survived at least the first 28 days on treatment, which was the primary endpoint of the trial. Immediate start of ruxolitinib after deterioration was associated with improved outcome, as was a lymphocyte-to-neutrophils ratio above 0.07. Together, treatment with the janus-kinase inhibitor ruxolitinib is feasible and might be efficacious in COVID-19 induced ARDS patients requiring invasive mechanical ventilation. The trial has been registered under EudraCT-No.: 2020-001732-10 and NCT04359290.

## Introduction

The novel coronavirus, SARS-CoV-2 [1], has created a dramatic global health and economic crisis. COVID-19 is the disease caused by SARS-CoV-2. In most cases, COVID-19 is associated with mild respiratory symptoms. However, in ~15% of the patients, hospitalization is required, and about 5% of patients develop severe lung injury including acute respiratory distress syndrome (ARDS). ARDS may be accompanied by sepsis and septic shock, and multiorgan failure. Older age, obesity, pulmonary and other comorbidities are risk factors for higher mortality [2, 3].

It has also been reported that the extent of inflammation—mirrored by peripheral blood cytokine levels—is associated with an inferior outcome [4]. In line with this, patients with severe COVID-19 disease benefit from immune-suppressive therapy with dexamethasone [5]. In this large trial, dexamethasone reduced day 28 mortality in patients with respiratory support at the time of randomization by 36%. Other immunosuppressants such as baricitinib (targeting janus-kinases; in combination with remdesivir) [6], or tocilizumab (targeting IL6) have also been tested in severe COVID-19 disease [7, 8], but have not changed clinical practice yet (reviewed in Ghosn et al. [9]). Interestingly, induction of neutrophil extracellular traps (NET) may contribute to severe COVID-19 by immunothrombosis [10].

That inflammatory pathways such as interferon-signaling play a key role in the disease progression has recently been shown by genome-wide association studies [11]. Ruxolitinib is an inhibitor of janus-kinases 1 and 2 [12, 13] that play a key role in inflammation. It is approved for the treatment of polycythemia vera and myelofibrosis, and also shows favorable immunosuppressive effects in steroid-refractory graft versus host disease (GvHD) in patients after allogeneic stem cell transplantation [14, 15]. Ruxolitinib has been used in patients with non-severe hyperinflammatory COVID-19 (see La Rosée et al. [16], for example). In keeping, clinical trials have shown that ruxolitinib may be efficacious in COVID-19 [17–19]. In a randomized phase III trial, ruxolitinib failed to show an overall survival benefit in patients with COVID-19 (NCT04362137). However, patients in this trial were not severely ill. As the SARS-CoV-2 mediated hyperinflammation is a complication that is associated with excess mortality at a progressed stage of the COVID-19 disease, we reasoned that janus-kinase inhibition using ruxolitinib would be rational in mechanically ventilated patients with COVID-19-associated ARDS.

Here we report the results of a single arm phase II study using ruxolitinib in patients with COVID-19 induced ARDS requiring mechanical ventilation. The primary endpoint, survival at day 28, was reached by 13/16 included patients (81%); this number compares favorably with other case series and publications.

## Patients, materials and methods

### Patients

Patients were eligible for this trial if they suffered from nucleic-acid-based test proven COVID-19 disease and associated severe lung injury as defined by recent intubation; requirement of invasive ventilation (NIV failure; respiratory rate > 30 and PaO2/FiO2 < 200 mmHg); moderate to severe pulmonary oxygen exchange disturbance as defined by (PaO2/FiO2) ≤ 200 mmHg at a PEEP ≥ 5 mm H2O; serum LDH > 283 U/l; ferritin above normal value and a CT-scan demonstrating COVID-19 typical pulmonary infiltrates. Patients or their representatives/legal person in charge had to give informed consent.

The primary endpoint was overall survival at day 28 after commencing ruxolitinib. Secondary endpoints included overall survival at 90 days after start of ruxolitinib; assessment of the duration of ventilation support; assessment of the extent of cytokine reduction (IL-6, CRP, ferritin); time on ICU; toxicity as well as safety and toxicity of ruxolitinib treatment as assessed by CTCAE criteria for grading (version 5.0).

The study protocol has been approved by German authorities (BfArM) and the local ethics committees at each site. Between July 07th and October 28th 2020, 18 patients were recruited at three German institutions (UKGM, Campus Marburg, Klinikum Kassel; University Hospital Aachen). Of the 18 patients that were screened, 16 patients were eligible for the trial (Supplementary Fig. 1; Consort diagram). Ruxolitinib was commenced at a dose of 2 × 10 mg (this was later changed to 2 × 10 − 15 mg, however, as part of an amendment), and dose increased to 2 × 15 mg from d2 to 28.

The drug was dissolved in water and administered via nasogastral tube. Novartis Pharma supported the trial and provided study drug and funding. Trial sponsor was the Coordinating Center for Clinical Trials at the Philipps University Marburg.

### Polymerase-chain-reaction

#### PCR

Diagnosis of SARS-CoV-2 infection was proven by combined E- and S-specific PCR (RealStar^®^ SARS-CoV-2 RT-PCR Kit, Altona Diagnostics, Hamburg, Germany) from a nasopharyngeal swab.

### Statistics

A sample size of *N* = 15 was chosen in order to assess potential feasibility of a subsequent randomized controlled trial. Overall survival was estimated using the Kaplan–Meier method. In post-hoc analyses, two-sided Wilcoxon-Mann–Whitney test was applied to compare distributions of C-reactive protein (CRP), Interleukin-6 (Il-6), and Ferritin levels at each assessment day against baseline, as well as durations of invasive ventilation before treatment started, and lymphocyte-neutrophil ratios (LNR) at baseline according to patient outcomes. Statistical results reported for exploratory (post-hoc) endpoints have not been corrected for multiple testing and *p* values therein are of descriptive nature.To compare event time distributions between patients according to LNR baseline levels, a log-rank test was applied as a further post-hoc analysis comparing two groups of patients with LNR at baseline above the median and those below or equal. Treatment exposure was summarized, including the average dose received by each patient, and the cumulative dose. All analyses are based on the intention-to-treat population and were performed using SAS (version 9.4 M3). For creation of figures, ggplot2 [20] for the R environment [21] was used.

### Data sharing agreement

Anonymized individual patient data will be made available to the scientific community with as few restrictions as feasible. However, until the publication of major outcomes, exclusive use will be retained. Data requests to be considered by the trial steering committee should be submitted by qualified researchers to AN (neubauer@staff.uni-marburg.de).

## Results

### Patients

Of the 18 screened patients, sixteen were eligible and received ruxolitinib as per protocol (Supplementary Fig. 1). Demographic data of the 16 patients on mechanical ventilation are displayed in Table 1. Median age of the study cohort was 59 years (range 35–92), and 13/16 (81%) were male. Median BMI was 28.0 (range 22–47.8). All 16 patients received dexamethasone according to the Oxford trial [5].

**Table 1:** Baseline characteristics in the intention-to-treat population (*N* = 16; all hospitalized and on invasive ventilation).

	*N*	Mean (±SD)	Median or freq.	Range or percentage
Age (yrs.)	16	59.5 (±14.92)	59	35–92
Sex, female/male (Pts./%)	16		3/13	18.75/81.25%
Weight (kg)	16	89.25 (±17.76)	90	60–127
Height (cm)	16	175.2 (±6.06)	175	163–186
BMI (kg/m^2^)	16	29.2 (±6.65)	28	22–47.8
*Vital signs*				
Heart rate (beats per minute)	16	77.1 (±17.1)	74.0	55–120
Respiratory rate (breaths per minute)	16	16.8 (±5)	14.5	12–28
PaO2/FiO2 (ratio)	16	167.3 (±148.7)	147.5	55–680
Blood Pressure, systolic (mmHg)	16	109.8 (±13.2)	110.0	90–135
Blood Pressure, diastolic (mmHg)	16	55.4 (±10.7)	55.0	40–80
Temperature (°C)	16	36.8 (±1.3)	36	35–39.6
*Risk factors*				
	**N Pts**.			**N Risk Factors**
Chronic liver disease	2			2
Chronic lung disease	2			2
Chronic nervous system disorder	3			3
DNR status	1			1
Diabetes	4			4
Disorder of cardiovascular system	10			20
Malignant neoplastic disease	2			2
Respiratory therapy (before trial)	12			12
Rheumatological/Immunological disease	4			7
Smoking status (smoker/former smoker)	6			6
Thromboembolic event	3			3

### Overall survival

**Fig. 1:**
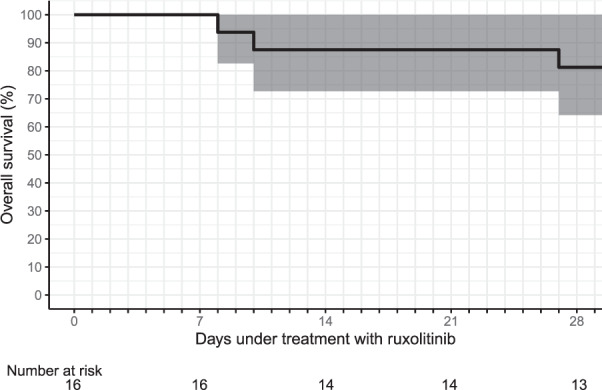
Kaplan–Meier estimated 28-day overall survival in the intention-to-treat population (*N* = 16). Gray denotes 95% pointwise confidence intervals (as based on the log hazard).

Thirteen of 16 patients in total enrolled in the trial were alive at day 28, resulting in an overall survival of 81% (Fig. 1).

### Secondary outcomes

**Fig. 2:**
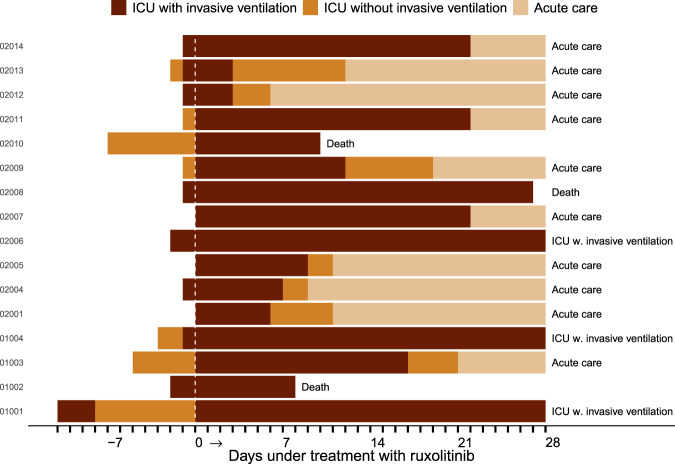
Condition of all patients during ICU treatment. Individual patient outcomes and times on ICU with the duration of invasive mechanical ventilation in the intention-to-treat population are shown (*N* = 16).

The average admission on ICU over the treatment period of 28 days was 16 days (median; range: 6–28 days) with an average duration of invasive ventilation support of 14.5 days (median; range: 3–28 days). The swimmers plot indicates that some patients were only treated on the ICU for a rather short period of time; this was also true for invasive mechanical ventilation in some patients (Fig. 2). Three patients received extra-corporal membrane oxygenization (ECMO) over a period of 10 days (*n* = 1) or over the total 28 days (*n* = 2).

**Fig. 3:**
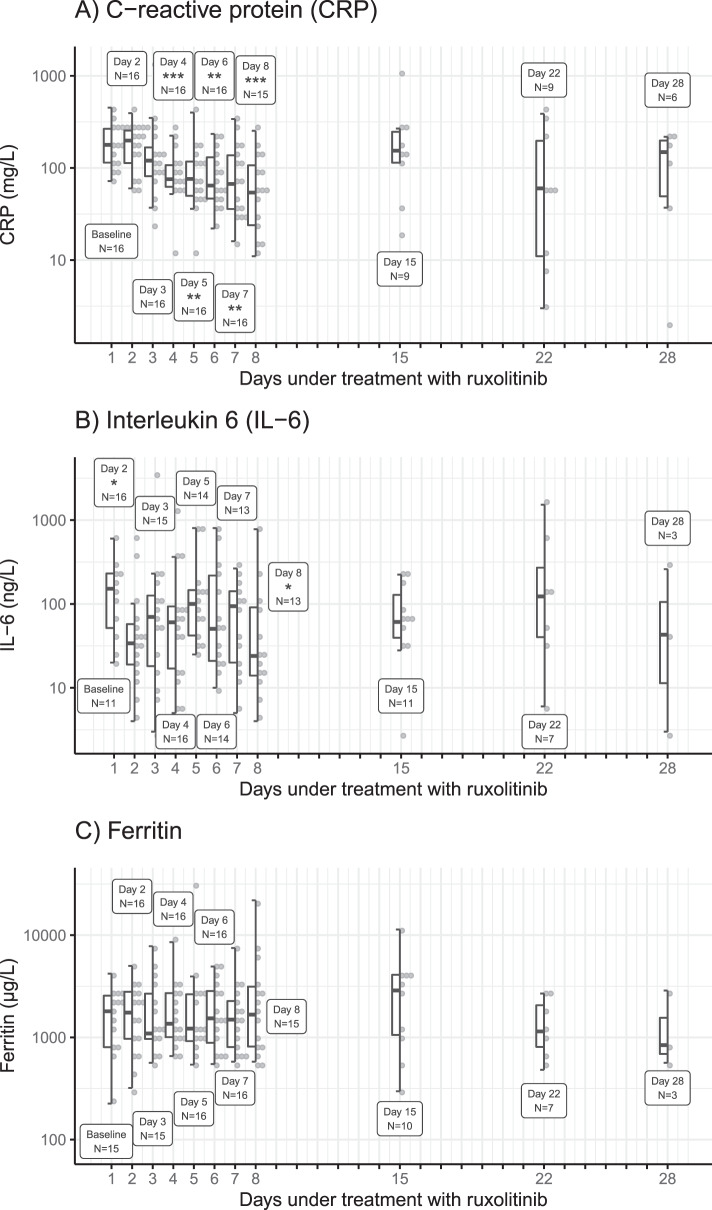
Biomarkers during ruxolitinib treatment. C-reactive protein (panel A), interleukin-6 (panel B), and ferritin (panel C) levels over 28 days treatment period in the intention-to-treat population (*N* = 16) with corresponding numbers of patients observed at each assessment. Note that the *y* axes are log-scale. Asterisks show significance level of *p* value obtained in two-sided Wilcoxon-Mann–Whitney test for a given assessment when compared to baseline distribution (*P < 0.05; **P < 0.01; ***P < 0.001). Please note, that these values have not been corrected for multiple testing.

Another secondary endpoint was the determination of inflammatory parameters such as C-reactive protein (CRP) or interleukin-6, respectively. Here, levels of CRP as well as interleukin-6 decreased significantly during treatment, in contrast to ferritin (Fig. 3).

### Treatment duration and side effects

Average treatment duration of patients receiving ruxolitinib was 20 days (median, range: 4–28 days). Treatment was interrupted in two patients due to adverse events (8 days interruption) and non-availability of a gastric tube (2.5 days interruption). Reasons for end of treatment before completion of all planned medications as scheduled (i.e., after 28 days) were: patients being discharged from ICU (*n* = 4) or transferred to another hospital (*n* = 3), patient death (*n* = 3), or adverse events (*n* = 2). Average dose intensity was 92.9% (median). Taking into account one patient with an average dose intensity below 80% (72.5%) results in an overall adherence rate to the protocol-specified dose in 15/16 patients (93.8%). The median cumulative ruxolitinib dose per patients was 530 mg (range: 105–810 mg). Ruxolitinib was generally well tolerated. As is the case for COVID-19 patients being mechanically ventilated, complications of the underlying disease occur frequently which can be mistaken for side effects. Table 2 lists adverse events (Severity 3), and Supplementary Table 1 shows serious adverse events of all patients.

**Table 2:** Adverse events (Severity 3) in patient population (total).

Preferred Term	*N*	%	Cumulative Frequency (*N*)
Dialysis	3	18.75	3
Aspartate aminotransferase increased	2	12.5	5
Activated partial thromboplastin time prolonged	1	6.25	6
Alanine aminotransferase increased	1	6.25	7
Anaemia	1	6.25	8
Blood bilirubin increased	1	6.25	9
Face oedema	1	6.25	10
Facial nerve disorder	1	6.25	11
Haematoma evacuation	1	6.25	12
Haemothorax	1	6.25	13
Hepatic enzyme increased	1	6.25	14
Leg amputation	1	6.25	15
Leukocytosis	1	6.25	16
Plasma protein metabolism disorder	1	6.25	17
Pneumonia	1	6.25	18
Pulmonary haemorrhage	1	6.25	19
Sepsis	1	6.25	20
Serum ferritin increased	1	6.25	21
Thrombin time prolonged	1	6.25	22
Troponin I increased	1	6.25	23

In one patient, life-threatening pneumonia improved to grade 3 pneumonia. In case of Ferritin, grade 3 investigations have been defined as “severe or medically significant but not immediately life-threatening” (as in the applicable CTCAE grading v5.0 section “Investigations-other”).

### Start of treatment may be critical

The time point of start of ruxolitinib was critical for outcome. Patients either being still on ICU with invasive ventilation or having died after 28 days were ventilated more than one day longer, on average (difference in medians is 1.5, difference in means is 1.1), before treatment with ruxolitinib had started, as compared to patients who were on acute care at the end of their treatment period after 28 days (*P* = 0.03; Wilcoxon-Mann–Whitney test).

### Lymphocytes-neutrophils ratio

**Fig. 4:**
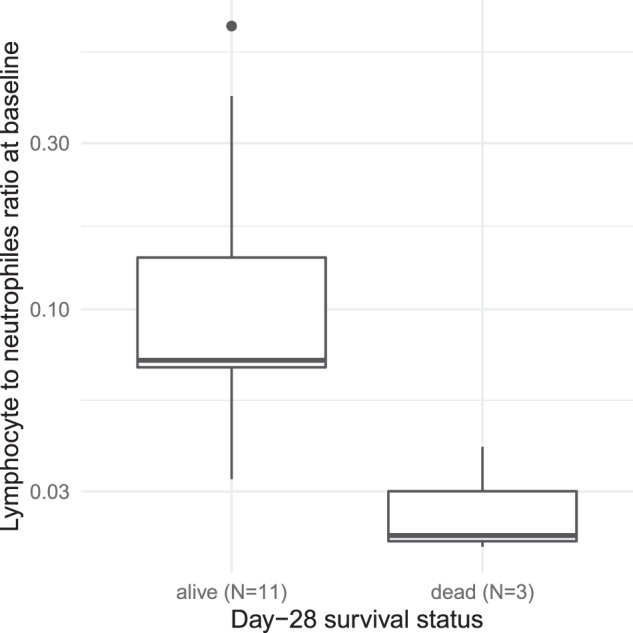
Lymphocyte to neutrophile ratio at baseline (log-scale) in day-28 survivors (*N* = 11) and non-survivors (*N* = 3). Note that the *y* axis is log-scale. Only 14 patients were evaluable as one leukemic patient had to be excluded, and in another patient no data were available. *P* < 0.01 (Exact Wilcoxon-Mann–Whitney test).

In this trial, several laboratory parameters were determined at study entry in addition to the ones displayed in Fig. 3. One of the most intriguing was the lymphocytes-neutrophils ratio (LNR) as determined by routine laboratory assessments. Mortality was associated with lower LNR at baseline, since all three patients who died before day 28 showed reduced LNR at baseline (Fig. 4). In the log-rank test the survival difference between patients having LNR at baseline greater or equal to average (median: 0.07) and those patients whose LNR at baseline was below average was estimated at *p* = 0.05 (Chi-Square = 3.9 on 1 degree of freedom) (not shown).

## Discussion

Since its first description [1], the novel coronavirus SARS-CoV-2 has caused a dramatic threat to global health and economy [22]. COVID-19 is the disease caused by SARS-CoV-2. SARS-CoV-2 is derived from SARS-CoV-1 and MERS; in most patients, infection does not cause life-threatening disease. SARS-CoV-2 replicates in the upper airway system, and can cause severe multisystem inflammatory reactions. In some patients, COVID-19 results in life-threatening acute respiratory distress syndrome ARDS, that is characterized by a prolonged clinical course. Several authors have investigated potential risk factors for death in COVID-19 (see [23–25]).

We and others have reported that blockade of Janus-kinases using ruxolitinib may be beneficial in COVID-19 [16–19, 26, 27]. However, a recently reported randomized clinical trial using ruxolitinib in non-severe COVID-19 (Ruxcovid) has not shown a beneficial effect (NCT04362137). We reasoned that inhibiting janus-kinases would especially be beneficial in severe COVID-19, mainly in patients suffering from ARDS. In these patients, the cytokine-storm is thought to be responsible for end-stage organ damage. Therefore, we performed a single arm phase II prospective clinical trial using ruxolitinib plus standard of care in mechanically ventilated ARDS patients suffering from COVID-19.

In our study including 16 patients, survival after 28 days was 81% (13/16). This number compares favorably to the range between 60 and 25% seen in previously reported day 28 survival rates for COVID-19 ARDS patients [28–30]. Of course, as the number of treated patients in our trial was small, and as this was a single-arm trial, it is too early to draw definitive conclusions as to whether ruxolitinib may be beneficial in COVID-19 ARDS patients. The janus-kinase inhibitor baricitinib, in combination with remdesevir, has shown improved survival in COVID-19 patients [6]. In keeping, in our patients, immediate start of ruxolitinib therapy seemed critical in that patients that died or were still hospitalized at day 28 revealed longer time on ventilation (*p* = 0.03). Ruxolitinib is known to suppress the function of CD4 positive T-cells in general [31]. It has also been reported that, for this T-cell inhibitory capacity, inhibition of JAK1 seems more important as compared to JAK2, and that STAT1 is responsible to execute this immunomodulation by JAK-inhibition [32]. In line with this, different JAK-inhibitors may yield in different side effects with regard to their sub-specificities [33]. As ruxolitinib inhibits both JAK1 and JAK2, it may be a rather promising candidate to be used in COVID-19 induced hyperinflammatory diseases. Another interesting note comes from a recent publication on the role of ruxolitinib in patients suffering from myeloproliferative neoplasms (MPN; where ruxolitinib is approved); here, patients discontinuing ruxolitinib had a dismal outcome as compared to MPN-patients continuing ruxolitinib [34]. Another drug, ibrutinib, that inhibits Bruton-kinase, may protect against pulmonary injury in COVID-19 [35]. Interestingly, very recently, interleukin-6 antibodies tocilizumab and sarilumab have shown to improve outcome in COVID-19 patients under mechanical ventilation [36]. Only a randomized clinical trial can thus prove that ruxolitinib may be of benefit in these patients.

Of note, survival in our small trial was improved when patients showed a lymphocytes- neutrophiles ratio (LNR) at study entry of at least 0.07 as compared to patients with a LNR below 0.07; all patients deceased before day 29 (*N* = 3) showed LNR levels below median at baseline, i.e., had LNR values below total median of 0.07 after exclusion of one leukemic patient (see Fig. 4). It has been published also by others that LNR is prognostic in severe COVID-19 (for example: [37]). Thus, ruxolitinib was not able to counteract upon this.

Taken together, ruxolitinib may be an effective drug in COVID-19 ARDS patients; however, a randomized clinical trial is mandatory to prove this. A trial investigating this question is underway (see: NCT04377620).

## Supplementary information

Supplemental Material
