# Supplementary material for: The janus-kinase inhibitor ruxolitinib in SARS-CoV-2 induced acute respiratory distress syndrome (ARDS)
Source: Leukemia. 2021 Aug 12;35(10):2917–23. doi: 10.1038/s41375-021-01374-3 (PMC8358255; doi:10.1038/s41375-021-01374-3)
Supplement: Supplementary file 1 — Supplemental Material [file 41375_2021_1374_MOESM1_ESM.pdf]

SUPPLEMENTAL APPENDIX

The janus-kinase inhibitor ruxolitinib in SARS-CoV-2 induced acute respiratory distress syndrome (ARDS)

Andreas Neubauer et al.

Contents

Supplemental Figure 1: Patient Disposition Chart 2
Supplemental Table 1: Serious adverse events in patient population with grade of severity .. 3

Supplemental Figure 1: Patient Disposition Chart

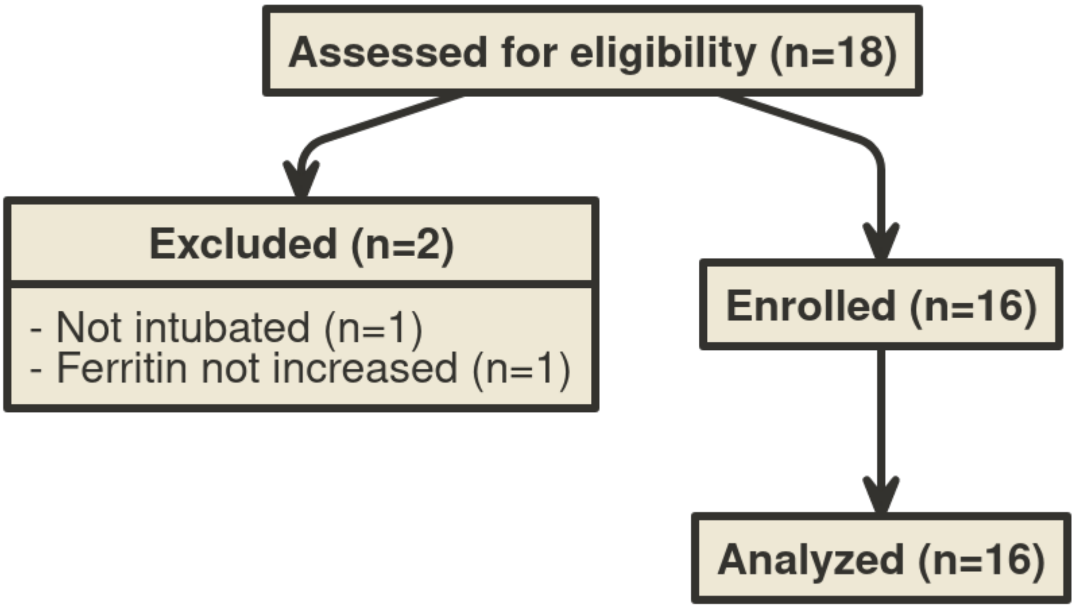

Suppl. Figure 1: Consort diagram.

Supplemental Table 1: Serious adverse events in patient population with grade of severity

Patient ID	Date of onset	Preferred Term	Drug Name	Causality	Severity
1001	21OCT2020	Pulmonary sepsis	Ruxolitinib	No reasonable possibility	Severe
1002	29OCT2020	Cardiac arrest	Ruxolitinib	No reasonable possibility	Severe
1002	24OCT2020	Sepsis	Ruxolitinib	No reasonable possibility	Severe
1003	10NOV2020	Alanine aminotransferase increased	Ruxolitinib	Reasonable possibility	Severe
1004	29OCT2020	Hypotension	Ruxolitinib	No reasonable possibility	Severe
1004	26OCT2020	Multiple organ dysfunction syndrome	Ruxolitinib	No reasonable possibility	Severe
1004	04NOV2020	Septic shock	Ruxolitinib	No reasonable possibility	Severe
2006	29OCT2020	Hypoxia	Ruxolitinib	No reasonable possibility	Severe
2008	08NOV2020	Cerebral venous sinus thrombosis	Ruxolitinib	No reasonable possibility	Severe
2008	13NOV2020	COVID-19 pneumonia	Ruxolitinib	No reasonable possibility	Severe
2008	17OCT2020	Peripheral artery thrombosis	Ruxolitinib	No reasonable possibility	Severe
2009	20DEC2020	Death	Ruxolitinib	No reasonable possibility	Severe
2010	29OCT2020	Respiratory failure	Ruxolitinib	No reasonable possibility	Severe
2011	21NOV2020	Multiple organ dysfunction syndrome	Ruxolitinib	No reasonable possibility	Severe
